# Measuring Daily Compliance With Physical Activity Tracking in Ambulatory Surgery Patients: Comparative Analysis of Five Compliance Criteria

**DOI:** 10.2196/22846

**Published:** 2021-01-26

**Authors:** Ryan Kelly, Simon Jones, Blaine Price, Dmitri Katz, Ciaran McCormick, Oliver Pearce

**Affiliations:** 1 School of Computing and Information Systems University of Melbourne Melbourne Australia; 2 Department of Computer Science University of Bath Bath United Kingdom; 3 School of Computing and Communications The Open University Milton Keynes United Kingdom; 4 Trauma and Orthopaedics Department Milton Keynes University Hospital Milton Keynes United Kingdom

**Keywords:** activity tracking, adherence, compliance, surgery, total knee arthroplasty

## Abstract

**Background:**

Physical activity trackers such as the Fitbit can allow clinicians to monitor the recovery of their patients following surgery. An important issue when analyzing activity tracker data is to determine patients’ daily compliance with wearing their assigned device, using an appropriate criterion to determine a valid day of wear. However, it is currently unclear as to how different criteria can affect the reported compliance of patients recovering from ambulatory surgery. Investigating this issue can help to inform the use of activity data by revealing factors that may impact compliance calculations.

**Objective:**

This study aimed to understand how using different criteria can affect the reported compliance with activity tracking in ambulatory surgery patients. It also aimed to investigate factors that explain variation between the outcomes of different compliance criteria.

**Methods:**

A total of 62 patients who were scheduled to undergo total knee arthroplasty (TKA, ie, knee replacement) volunteered to wear a commercial Fitbit Zip activity tracker over an 8-week perioperative period. Patients were asked to wear the Fitbit Zip daily, beginning 2 weeks prior to their surgery and ending 6 weeks after surgery. Of the 62 patients who enrolled in the study, 20 provided Fitbit data and underwent successful surgery. The Fitbit data were analyzed using 5 different daily compliance criteria, which consider patients as compliant with daily tracking if they either register >0 steps in a day, register >500 steps in a day, register at least one step in 10 different hours of the day, register >0 steps in 3 distinct time windows, or register >0 steps in 3 out of 4 six-hour time windows. The criteria were compared in terms of compliance outcomes produced for each patient. Data were explored using heatmaps and line graphs. Linear mixed models were used to identify factors that lead to variation between compliance outcomes across the sample.

**Results:**

The 5 compliance criteria produce different outcomes when applied to the patients’ data, with an average 24% difference in reported compliance between the most lenient and strictest criteria. However, the extent to which each patient’s reported compliance was impacted by different criteria was not uniform. Some individuals were relatively unaffected, whereas others varied by up to 72%. Wearing the activity tracker as a clip-on device, rather than on the wrist, was associated with greater differences between compliance outcomes at the individual level (*P*=.004, *r*=.616). This effect was statistically significant (*P*<.001) in the first 2 weeks after surgery. There was also a small but significant main effect of age on compliance in the first 2 weeks after surgery (*P*=.040). Gender and BMI were not associated with differences in individual compliance outcomes. Finally, the analysis revealed that surgery has an impact on patients’ compliance, with noticeable reductions in activity following surgery. These reductions affect compliance calculations by discarding greater amounts of data under strict criteria.

**Conclusions:**

This study suggests that different compliance criteria cannot be used interchangeably to analyze activity data provided by TKA patients. Surgery leads to a temporary reduction in patients’ mobility, which affects their reported compliance when strict thresholds are used. Reductions in mobility suggest that the use of lenient compliance criteria, such as >0 steps or windowed approaches, can avoid unnecessary data exclusion over the perioperative period. Encouraging patients to wear the device at their wrist may improve data quality by increasing the likelihood of patients wearing their tracker and ensuring that activity is registered in the 2 weeks after surgery.

**Trial Registration:**

ClinicalTrials.gov NCT03518866; https://clinicaltrials.gov/ct2/show/NCT03518866

## Introduction

### Background

Commercial activity trackers hold great promise for enabling clinicians to understand the physical activity of patients following surgery. Devices such as Fitbit or Apple Smart Watch, which typically present physical activity as a daily step count [1-3], have been shown to provide clinically viable data that are more accurate than patients’ self-reports [4,5]. In light of these capabilities, researchers have explored how activity trackers can help to monitor patient recovery, particularly in cases involving ambulatory surgery [6,7], where early mobility is thought to result in better outcomes [8].

A necessary precursor to determining a patient’s activity level is compliance analysis [9,10]. The principal aim of compliance analysis is to determine whether a patient provided reliable data on any given day, within a defined tracking period. What counts as reliable data is determined by setting a threshold for inclusion and discarding data that do not meet the threshold. As an example, a patient might be instructed to wear a tracking device every day for a minimum of 8 hours and would be considered compliant on days when the protocol was followed [9]. Any noncompliant days are then excluded from the data set, ensuring that only valid days are used in subsequent analyses [10]. Calculating each patient’s compliance helps to ensure the validity, quality, and trustworthiness of activity tracking data [10-13]. Compliance analysis also supports reliable inferences based on the acquired data [14], including assessment of patient recovery [15,16] and activity levels [17].

A crucial step in compliance analysis involves selecting an appropriate criterion for data filtering. The literature harbors a range of criteria that differ in terms of how they define a valid day and hence filter data [10]. For example, quantity-based measures filter data based on absolute step counts [18], whereas time-based measures filter data based on criteria such as hours of device wear per day [19]. Compliance criteria also vary in their leniency. For instance, the criterion “>0 steps” considers a day as valid if at least one step is recorded within a 24-hour window [20]. Conversely, the criterion “≥10 hours” requires at least one step to be registered in 10 distinct hours over the day [21]. Problematically, the variety of available compliance criteria can leave researchers unsure which to use when working with activity data. It has also been shown that rates of data inclusion change when different compliance criteria are used to analyze an identical data set [10]. Strict criteria can often result in many otherwise valid days being discarded [10]. These issues motivate the need for researchers to investigate the impact of different compliance criteria when analyzing activity data.

In this work, we investigate the effect of applying different compliance criteria to activity data acquired from patients undergoing ambulatory surgery. We focus on total knee arthroplasty (TKA, ie, knee replacement) as it is a procedure that relates directly to movement and physical functioning [22]. TKA often results in pain and swelling [23], causing temporary reductions in mobility [24] that subside over time as functionality returns to the joint [6,7]. Tracking TKA patients’ activity is therefore valuable to clinicians [25] and commercial systems are being developed that help clinicians to monitor patient recovery by collecting activity data [26]. However, analyzing compliance in TKA patients is not straightforward because these patients often have limited mobility following surgery. Selecting an overly strict compliance criterion to filter data may therefore result in the unnecessary exclusion of days, simply because a patient was undergoing recovery. Moreover, a patient could be compliant with device usage without registering steps due to reduced mobility, leaving ambiguity as to whether the lack of steps indicates that the device was not worn or whether there was no movement. These issues point toward a need to understand how different compliance criteria can affect the analysis of activity data from TKA patients; how compliance analyses should account for fluctuations in mobility over the perioperative period; and how researchers should select an appropriate criterion for analyzing activity data from TKA patients, such that recorded data are not unnecessarily discarded on the basis of a low step count.

### Aims of This Study

This study aimed to understand the impact of applying 5 different compliance criteria to the activity tracking data of TKA patients. We further sought to investigate causes of variation in compliance outcomes through analysis of patients’ tracked data.

## Methods

### Setting

This study was a field study in which TKA patients were asked to wear a commercial Fitbit Zip activity tracker (Fitbit, Inc.) to record their everyday physical activity, in the form of step count, over an 8-week perioperative period. The perioperative period comprised the 2 weeks before the patient’s surgery, the day of surgery, and the 6-week postoperative period (57 days in total). The study was conducted in the United Kingdom as part of a planned clinical trial (Trial Registration No. NCT03518866) that explored associations between early mobility and patient-reported outcomes following TKA (not reported here). The trial involved calculating each patient’s compliance with wearing their assigned tracker. This task brought the effects of different compliance criteria to our attention and motivated this analysis.

### Ethics and Recruitment

The study occurred over a 3-year period at the Open University in collaboration with Milton Keynes University Hospital (MKUH), a large public hospital in the United Kingdom. All procedures received approval from the Open University’s Human Research Ethics committee (ID: HREC/2014/1635/Price/1), the R&D Department at MKUH, and the NHS Health Research Authority London (Surrey Borders Research Ethics Committee, Reference: 15/LO/0649).

Participants were recruited by the sixth author (OP), who identified patients from the hospital’s elective orthopedic operating list. Inclusion criteria were as follows: adults undergoing TKA, ability to speak and understand conversational English, no cognitive impairments (eg, dementia), and no medical conditions unrelated to the individual’s surgery that may affect pain levels or ability to participate (eg, severe neurological disorder, acute cancer, psychiatric disorder, or infections).

Patients who met the inclusion criteria were invited to participate during an outpatient appointment with their consultant. An information pack was provided for each patient to take away and read (Multimedia Appendix 1). The pack contained an overview of the study, a consent form, a prestudy questionnaire, and the contact details of the researchers. A member of the research team (BP or LS, see “Acknowledgments”) followed up with the patient via telephone 1 week after their consultant appointment. Those who indicated willingness to participate were asked to bring the signed consent form and completed questionnaire with them to a presurgery clinic.

### Participants

A total of 62 patients volunteered for the study; 12 participants were excluded from the study because their scheduled surgery was canceled. A further 30 individuals were removed, either due to technical issues related to the Fitbit or because they withdrew from the study.

The final sample comprised 20 participants (7 men and 13 women) who underwent ambulatory surgery and provided Fitbit data. The mean age of the participants was 64.5 years (SD 8.94; range 37-76). All had mean hospital stays of 2 days (range 1-4 days) following their surgery. Preoperative health was assessed using the ASA-PS (American Society of Anesthesiologists Physical Status) classification [27]. One patient was ASA 1, 18 patients were ASA 2, and 1 patient was ASA 3. This is broadly representative of typical comorbidities for a UK population requiring TKA, who may also have arthritis in other joints [28]. Medication use was not assessed as its heterogeneity was not thought to lend itself to analysis of physical activity following TKA.

### Materials and Data Collection Setup

Participants were provided with a commercial Fitbit Zip activity tracker. This tracker was among the most popular commercial activity trackers when our study began, and was chosen due to its simplicity, high durability, and validity for capturing step data [29-31]. Furthermore, the Fitbit Zip has a 90-day battery life, avoiding the need for patients to charge the device (a known contributor to noncompliance [32]).

Participants who took part before January 2017 (N=8) were given the Fitbit Zip with a clip-on housing and were advised to wear it on their clothing (eg, belt loop), waist band, or brassiere strap. Some participants informally reported forgetting to unclip the device from their clothing, so the remaining participants (N=12) were given a plastic housing for the Zip that allowed them to wear the tracker around their wrist.

To avoid the need for participants to manage the synchronization of data collection from the Fitbit [19], a set of custom-made ‘Fitboxes’ was created by the research team. Each Fitbox was designed to be plugged into a network port on the participant’s home router. The Fitbox was a custom 3D-printed plastic box containing a Fitbit wireless dongle attached to a Raspberry Pi computer. The computer ran a Python script that used the Galileo library [33] to capture data from the Fitbit without user intervention. The computer scanned for the patient’s Fitbit every 10 minutes and synchronized the data to Fitbit’s cloud server via Bluetooth. Data were then extracted from Fitbit’s service for hosting on our own server.

### Procedure

After each participant had consented to participate, a Fitbit Zip and Fitbox were delivered to their home by postal mail, arriving 2 weeks prior to their scheduled surgery. The participant was asked to plug the Fitbox into their internet router and begin wearing the Fitbit immediately.

Participants were requested to wear the Fitbit for the following 8 weeks, including their scheduled date of surgery. The data collection was periodically monitored by a member of the research team (BP) to check if there were any technical issues. In 2 cases a faulty Fitbox was replaced.

At the end of the 8-week period, participants were informed that they could stop using the Fitbit and were asked to bring the equipment to a scheduled appointment with their consultant. Participants were thanked and debriefed about the purpose of the research.

### Data Analysis

Minute-by-minute step counts for each patient’s perioperative period were acquired using the Fitbit API. The data were analyzed using scripts that applied the 5 different compliance criteria to each participant’s data to determine daily compliance statistics. An average compliance outcome over the perioperative period was then derived for each criterion, and for each participant. The data were subsequently plotted using heatmaps and line graphs to investigate periods of the day in which patients registered their steps. Statistical tests were performed using JASP to explore factors that explain differences between calculated rates of compliance. In this analysis, all data from each participant were included as our aim was to explore differences between rates of compliance under each criterion, and how these differences impact data retention.

### Compliance Criteria

The 5 compliance criteria in our study are listed in Table 1. These criteria were chosen because they represent plausible approaches to assessing compliance in TKA patients, and because they have been used extensively in the literature on activity tracking [10].

As Table 1 illustrates, each criterion defines a different threshold for what counts as a valid day. The first 2, *>0 steps* and *>500 steps*, are quantity-based measures that assess compliance based on absolute thresholds for step count. To be considered compliant on a given day, a person must register either at least one step or more than 500 steps, respectively. The criterion *>0 steps* is very lenient and may therefore be useful for analyzing data from low-mobility populations, but can be problematic as it assumes there is no error in data collection. Activity trackers often register data from hand or arm movements [34], meaning that even the smallest incidental movement could be mistaken for a valid day of wear. Criteria like *>500 steps* address this problem by setting a higher minimum requirement for what counts as a valid day, though this may be too high to capture data from surgery patients during their recovery period. Patients may in fact be compliant with device use, while still taking fewer than 500 steps per day after surgery.

The next threshold, ≥*10 hours*, requires a person to register at least one step in 10 different hours of the day. Previous work has noted that this criterion is often used in health informatics, but it is among the most stringent and can lead to high rates of data exclusion [10].

The final 2 criteria, *3-a-day* and *3-of-4 windows*, are time-based measures that consider a day as valid if the patient registers data in 3 predefined periods. In *3-a-day*, a patient must register at least one step in 3 windows anchored to the morning, afternoon, and evening [35]. In *3-of-4* windows, the day is broken down into 4 equal windows of 6 hours each, and a person is required to register data in at least three of them to be compliant [36]. Both of these criteria assess compliance based on continued wear over the course of a day. However, the *3-a-day* criterion contains windows of unequal size, which may be problematic when assessing the compliance of people who work unusual hours or rotated shift work. The *3-of-4* windows criterion addresses this limitation by allowing the patient to register data in a more flexible schedule.

**Table 1:** Definitions of compliance for the 5 criteria used in this study.

Compliance criterion	Definition	Example study
>0 steps	A day is considered valid if the tracker registered at least one step, ie, any data whatsoever.	Epstein et al [20]
>500 steps	A day is considered valid if the tracker registered more than 500 steps in a day.	Meyer et al [37]
≥10 hours	A day is considered valid if the tracker registered data in at least ten different 1-hour windows.	Sirard and Slater [21]
3-a-day	A day is considered valid if the tracker registered data in 3 predefined periods: 3 am to 11 am, 11 am to 3 pm, and 3 pm to 3 am.	Meyer et al [35]
3-of-4 windows	A day is considered valid if the tracker registered data in at least three of four periods: 12 am to 6 am, 6 am to 12 pm, 12 pm to 6 pm, and 6 pm to 12 am.	Barak et al [36]

## Results

### Analysis Overview

We first consider how the 5 criteria produce different compliance outcomes across the entire sample. We then consider how the 5 criteria affect rates of calculated compliance for individual patients. This allows us to interrogate whether the difference in compliance outcomes is consistent across the sample or whether certain patients are more affected by changing criteria. We then consider how variations between compliance calculations vary over the perioperative period, investigating the extent to which variation in compliance can be explained by demographic factors and whether the Fitbit was a clip-on or wrist-worn device.

### Impacts of the Compliance Criteria Across the Sample

Table 2 shows the mean compliance for each criterion, averaged across the 8-week perioperative period for all 20 participants. Compliance can range from 0.00 to 1.00, with an outcome of 1.00 indicating 100% compliance.

Table 2 reveals that there is a difference in compliance outcomes between the criteria. For example, >0 steps gives a mean compliance of 0.80 (SD 0.17) across the sample, whereas ≥10 hours is lower at 0.56 (SD 0.25).

The histograms in Table 2 illustrate the proportion of the sample with each compliance level, showing that different criteria result in a different distribution of outcomes. For example, the distribution of compliance rates across all patients when using >0 steps is skewed toward 1.0, whereas the distribution for the ≥10 hours criterion is skewed toward 0.2. These outcomes reflect the nature of the measures, that is, >0 steps is the most lenient criterion whereas ≥10 hours is the strictest [10]. They also illustrate how different criteria lead to different rates of data exclusion, that is, a greater number of days are considered as invalid under the strictest criterion.

**Table 2:** Descriptive statistics for calculations of daily compliance with activity tracking across the patient sample, using each of the 5 compliance criteria. The histograms illustrate the proportion of patients with each compliance level under that criterion.

Compliance criterion	Mean (SD)	Minimum	Maximum	Range	Histogram
>0 steps	0.80 (0.17)	0.40	1.00	0.60	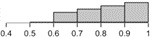
>500 steps	0.63 (0.22)	0.26	1.00	0.74	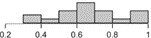
≥10 hours	0.56 (0.25)	0.02	0.96	0.94	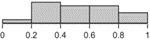
3-a-day	0.67 (0.22)	0.05	0.93	0.88	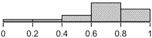
3-of-4 windows	0.67 (0.22)	0.04	0.93	0.89	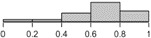

### Impacts of the Compliance Criteria Between Patients

**Figure 1:**
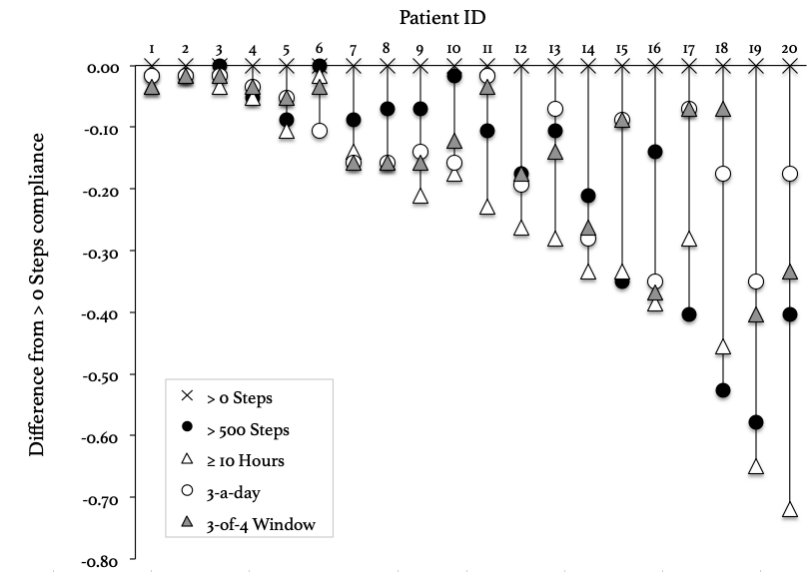
Differences in compliance calculations for the patients in our study. The scores are plotted against the most lenient criterion, >0 Steps, and show the difference between this criterion and the other measures.

Figure 1 provides a visual comparison of the compliance outcomes for each patient, showing the difference between >0 steps and the other 4 criteria. We use >0 steps as a baseline to illustrate the differences, as it produces the highest compliance outcome for all patients. In Figure 1, patients are listed according to the deviation between the compliance measures, ranging from smallest to greatest (left to right, respectively). Full data on each patient’s outcomes, along with demographic information, can be found in Multimedia Appendix 2.

We first consider differences between patients in terms of their calculated compliance with activity tracking, focusing on outcomes calculated *within* particular criteria. Overall, the data show that there are individual differences between patients in terms of calculated compliance. For example, 5 patients achieved 100% compliance under the >0 steps criterion, whereas patient 8 scored just 49%. Similarly, 3 patients had 98% or higher compliance under the >500 steps criterion, whereas others fared much worse. This reiterates evidence from the literature, that is, some people are highly compliant whereas others exhibit breaks or lapses in tracking [20].

We next consider differences between compliance criteria and how they change for each patient. A salient observation from Figure 1 is that the change produced from switching compliance criteria is not uniform across patients. Changing criteria can make minimal difference for some patients but has a substantial impact for others. For example, the outcomes for patients 1-4 are largely unaffected, whereas patient 20’s compliance moves from 100% valid days under >0 steps to just 28% under ≥10 hours. This equates to a 72% difference between the 2 criteria, meaning that much of the patient’s data would be excluded from further analysis under the stricter threshold.

Furthermore, we observed that changing criteria can *increase* reported compliance for some patients while *decreasing* reported compliance for others. This is illustrated by P16 and P17, and how their compliance changes when switching from >500 steps to ≥10 hours. P17 records a 12% *increase* in compliance when changing to ≥10 hours, whereas P16’s compliance *decreases* by 24%.

The observed disparity in the direction of change begs the question of why different compliance criteria result in changes between patients, and why some patients are more affected by changes than others. From examining our data, we observed an apparent trend whereby patients with wrist-worn devices appeared to exhibit less variation between compliance outcomes (ie, lower SD), whereas those with clip-on devices exhibit greater variation across the 5 criteria.

Statistical analysis of SD between compliance calculations using point biserial correlation revealed a statistically significant correlation (*r*=.616, *P*=.004). Hence, participants with a lower SD between compliance outcomes (ie, those toward the left side of Figure 1) were more likely to be using a wrist-worn Fitbit device than those with a higher SD (ie, those toward the right side of the graph, who were more likely to be wearing a clip-on device).

We also investigated whether age, BMI, and gender influenced compliance outcomes. Pearson correlation analysis revealed no significant relationship between age and SD of compliance outcomes (*r*=.178, *P*=.453). Likewise, Pearson correlation showed no significant relationship between BMI and SD (*r*=.147, *P*=.535). Point biserial correlation analysis revealed no significant relationship between gender and SD of compliance outcomes (*r*=.393, *P*=.086).

### Physical Activity Patterns Throughout the Perioperative Period

**Figure 2:**
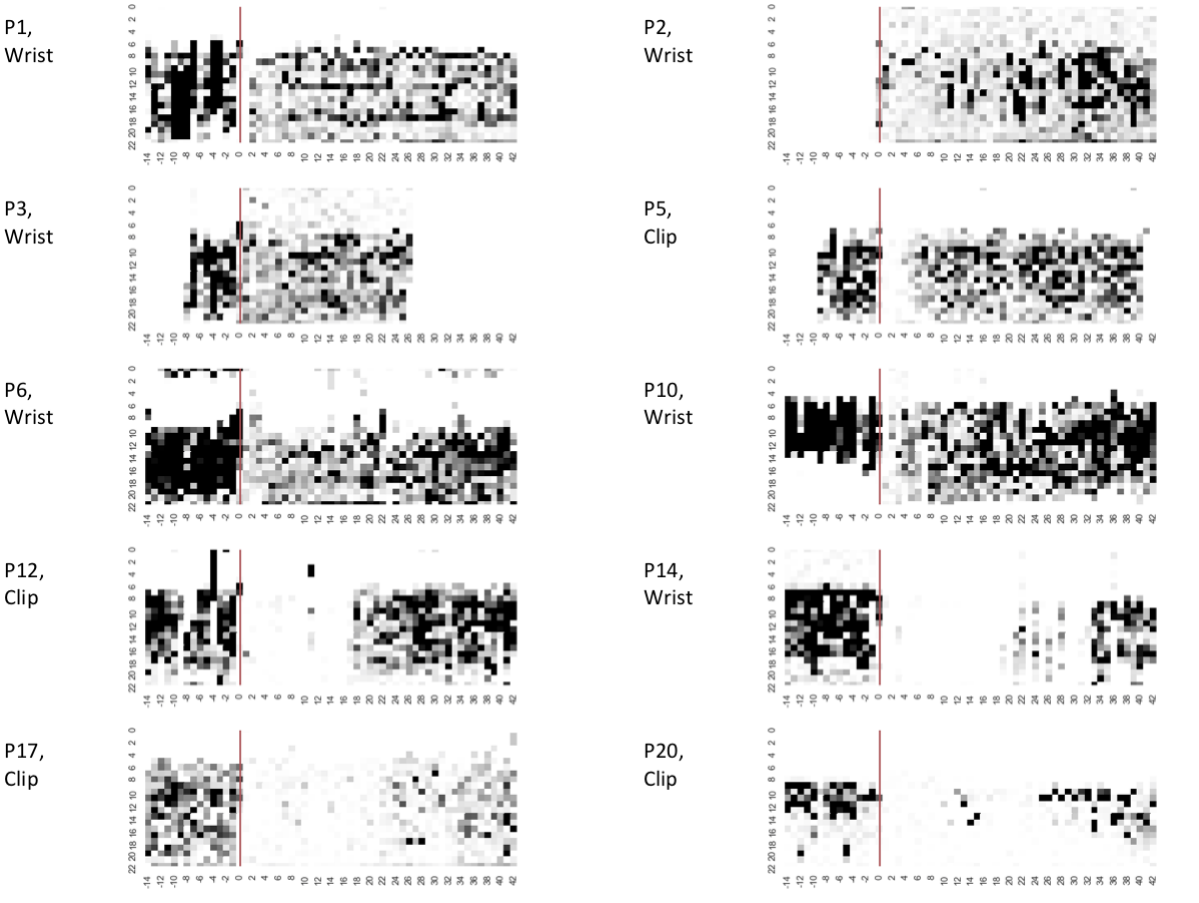
Heatmaps for 10 participants, illustrating the variations in patterns of physical activity throughout the day and across the 8-week perioperative period (from 2 weeks presurgery to 6 weeks postsurgery). The x-axis represents the day, beginning 2 weeks prior to surgery (day -14) and ending 6 weeks after surgery (day 42). The red line indicates the day of surgery (day 0). The y-axis represents the hour of the day. The color of cells corresponds to the number of steps recorded in a given hour, ranging from white (0) to black (500+).

To explore why the 5 criteria produce different compliance outcomes between patients, we generated heatmaps that visualize each patient’s raw step count data across the perioperative period. Figure 2 shows heatmaps for 10 patients in our sample who exhibit different patterns of activity. We selected these patients to illustrate how different activity patterns produce changes under different compliance measures. Heatmaps for all 20 patients are included in Multimedia Appendix 3.

The heatmaps resulted in 3 main observations.

First, there are general differences before and after surgery, with all of the participants exhibiting a noticeable change in their behavior after the red line, which indicates the day of surgery. Most patients (eg, P12, P14, and P17) exhibit a decline in the amount of data collected, whereas some others (such as P2) record more data. It is also notable that some patients have clear gaps in their data collection record. For example, P2 failed to record any data before the surgery, and P14 has many missing days following the surgery. These gaps are evidence of failing to wear the Fitbit and are considered as noncompliant days under all of the criteria in our analysis.

The second observation is that overall levels of recorded activity decrease after surgery among patients who appeared to be wearing their Fitbit during the preoperative period. For some patients this is illustrated by noticeable gaps in activity after surgery (eg, P5 and P12), whereas for others there is a simple reduction in the amount of data collected. Activity levels then typically increase over time, returning toward and sometimes exceeding a presurgery level, as illustrated by more dark cells appearing in the final weeks (eg, P2, P6, P12, and P17). This pattern is consistent with the gradual restoration of physical activity after TKA [6,7], but has implications for assessing compliance because the stricter criteria (eg, >500 steps and ≥10 hours) are likely to be insensitive to patients’ reduced activity following surgery.

A third observation is that some patients’ *distribution* of activity over the day remains the same following surgery, but for others it changes over time. For patients including P1, P5, P14, and P17, there is a continuation in the person’s distribution of activity from before and after surgery, even though the difference represents an overall reduction in the level of activity. By comparison, P10’s hours of activity end around 4 pm in the preoperative period but extend further into the evening after surgery.

The changes in activity level and distribution are important because they provide an explanation for why some patients may become gradually more compliant under certain criteria while remaining stable under others. In addition, the heatmaps suggest a clear impact of surgery on the patients’ activity level, providing a potential explanation as to why some individuals are considered as less compliant under stricter measures.

### Variations in Compliance Criteria Agreement Throughout the Perioperative Period

Our observations of the heatmap data highlighted temporal variations in patients’ recorded activity, with salient periods at which notable changes occur. The heatmaps also highlight specific periods of interest for which physical activity is likely to be altered, that is, immediately after surgery and throughout the recovery period. Thus, we investigated temporal variations in compliance criteria agreement, that is, how closely the 5 outcomes align with one another over time, and how this alignment varies throughout the perioperative period. This allows us to further understand the impact of surgery on compliance agreement across the sample.

**Figure 3:**
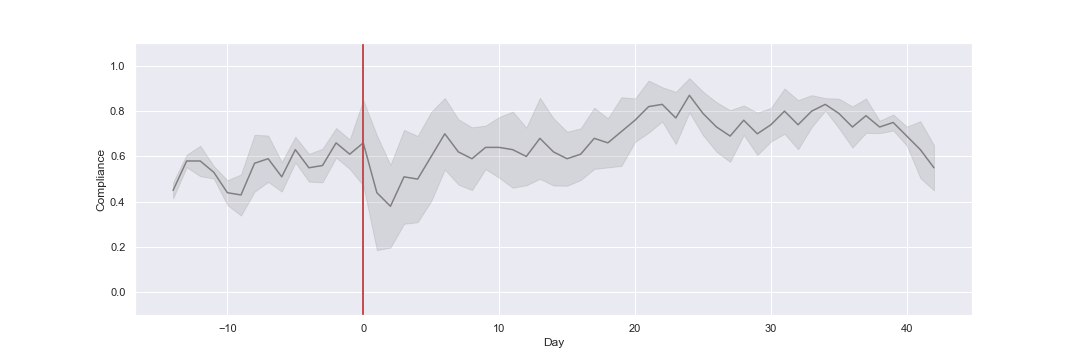
Daily compliance rates over time. The percentage of the sample that were compliant on any given day is illustrated by the y-axis. The dark gray line represents the mean average across all 5 of the compliance criteria. The height of the gray shaded region indicates the standard deviation between measures over time. The red vertical line represents the day of surgery.

Figure 3 shows the daily compliance rates for all 5 criteria, with the red line marking the day of surgery. The data suggest there was a trend toward higher average compliance toward the end of the perioperative period. There is a noticeable drop-off in mean compliance immediately following surgery, and a slight decrease around the 40-day mark.

In terms of agreement between the criteria, it can be seen that there is very little difference between measures in the presurgery phase, as reflected by the narrower shaded region for SD. After the surgery there is a 25% drop in the average compliance, with a corresponding increase in SD between criteria. Over the next 3 weeks, the mean increases while the SD decreases, as indicated by the shrinking height of the shaded area. Toward the end of the perioperative period, in the final 2 weeks, there is a dip in the average compliance but the deviation between measures is small and stable.

**Figure 4:**
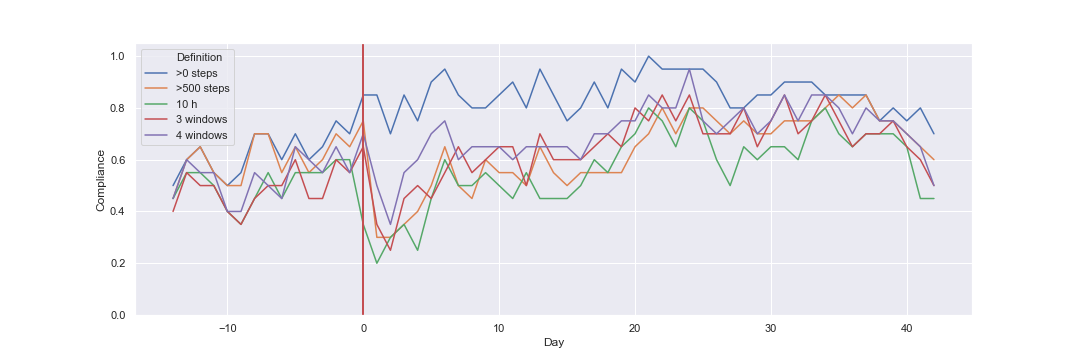
Comparison of daily compliance rates for all 5 compliance criteria.

Figure 4 shows specifically how patients’ compliance appears to vary over time when applying each of the criteria. There are several important observations from this figure. The first is that compliance under the >0 steps criterion is generally higher than the other criteria following surgery, with a very noticeable difference for the first 20 days of the recovery period. This means that there are many instances in which patients were registering at least some steps over the day, but the total number was often insufficient to be considered compliant on stricter thresholds.

The second main observation from Figure 4 is that the >500 steps, ≥10 hours, 3-a-day, and the 3-of-4 window thresholds appear to follow each other reasonably closely over the entire 8-week period. However, these 4 thresholds cause a large drop in calculated compliance after surgery, and then show a gradual increase over time. The implication of this is that the patient is registering a small number of steps in the days after his/her surgery, but those steps are not sufficient to be considered compliant under stricter activity thresholds.

A final observation is that there is a slight increase in compliance at the point of surgery under the >0 steps criterion. This likely indicates fewer cases of participants forgetting to wear the Fitbit, and could be caused by hospital staff reminding participants to wear the device, or by the fact that TKA may make the idea of monitoring physical activity more salient. Alternatively, surgery presents a change to routine and thus there may be fewer daily activities that are distracting from wearing the tracker.

Taken together, Figures 3 and 4 illustrate the plausible impact of temporary impairment on participants’ compliance under different criteria. Many days appear to be considered as valid under the most lenient criterion (>0 steps) but would be discarded under the other measures.

#### Analysis of Mean Calculated Compliance

To explore the preceding observations quantitatively, we subdivided the perioperative period into distinct 2-week stages (excluding the day of surgery) and calculated 2-week compliance figures for each patient, using the 5 criteria. This allowed us to explore whether there are differences between the 2-week stages (defined as *presurgery, weeks 0-2, weeks 2-4*, and *weeks 4-6*). We then used linear mixed models to investigate changes in calculated compliance between the 5 criteria, using demographic information as independent variables. Each patient’s average compliance for the 2-week stages is shown in Multimedia Appendix 4.

Specifically, our model compared the effects of *compliance criteria, gender*, *age*, *BMI*, *stage*, and *device type* (*clip or wrist-worn tracker*) on *2-week compliance*. ASA was excluded because there was insufficient variance in scores between participants. In each analysis the independent variables were set as fixed effects. Participant ID was set as a random effect. The reported β estimates indicate the value by which 2-week compliance varies for the respective condition. We report the standard error (SE) for the estimates and test for significance at α=.05.

We found a significant main effect of *compliance criteria* on *2-week compliance* (*F*_4,22.51_=16.816, *P*<.001). Compliance scores were significantly higher (approximately 16%) for >0 steps (β=.157, SE=0.022, *P*<.001). Compliance scores were significantly lower for >500 steps (β=–.041, SE=0.017, *P*=.027), ≥10 hours (β=–.104, SE=0.019, *P*<.001), and 3-a-day (β=–.031, SE=0.014, *P*=.045).

We also found a significant main effect of *stage* on *2-week compliance* (*F*_3,18_=4.166, *P*=.021). Compliance scores were significantly higher (approximately 10%) in the week 4-6 postsurgery *stage* (β=.102, SE=0.041, *P*=.024). This matches our observations of Figures 3 and 4, and indicates that patients were gradually regaining mobility and recording more data as the perioperative period progressed.

We also found a small but significant main effect of *age* on *2-week compliance* (*F*_1,15.48_=5.004, β=.009, SE=0.004, *P*=.040). Specifically, older patients were more likely to have higher compliance, regardless of criteria. This may be because health issues are more salient for older adults [38], potentially increasing their compliance with using an activity tracker.

Finally, we found significant interaction effects between *criteria* and *stage* (*F*_12,245.89_=5.631, *P*<.001). Compliance scores were significantly higher for the >0 steps criterion in weeks 0-2 postsurgery (β=.115 , SE=0.019, *P*<.001). Compliance scores were significantly lower for >500 steps (β=–.067, SE=0.019, *P*<.001) and 10 hours (β=–.054 , SE=0.019, *P*=.005) in weeks 0-2 postsurgery. Aligning with Figures 3 and 4, this outcome demonstrates that the weeks following surgery are associated with reductions in compliance under stricter criteria.

#### Variations Over Time by Device Type

**Figure 5:**
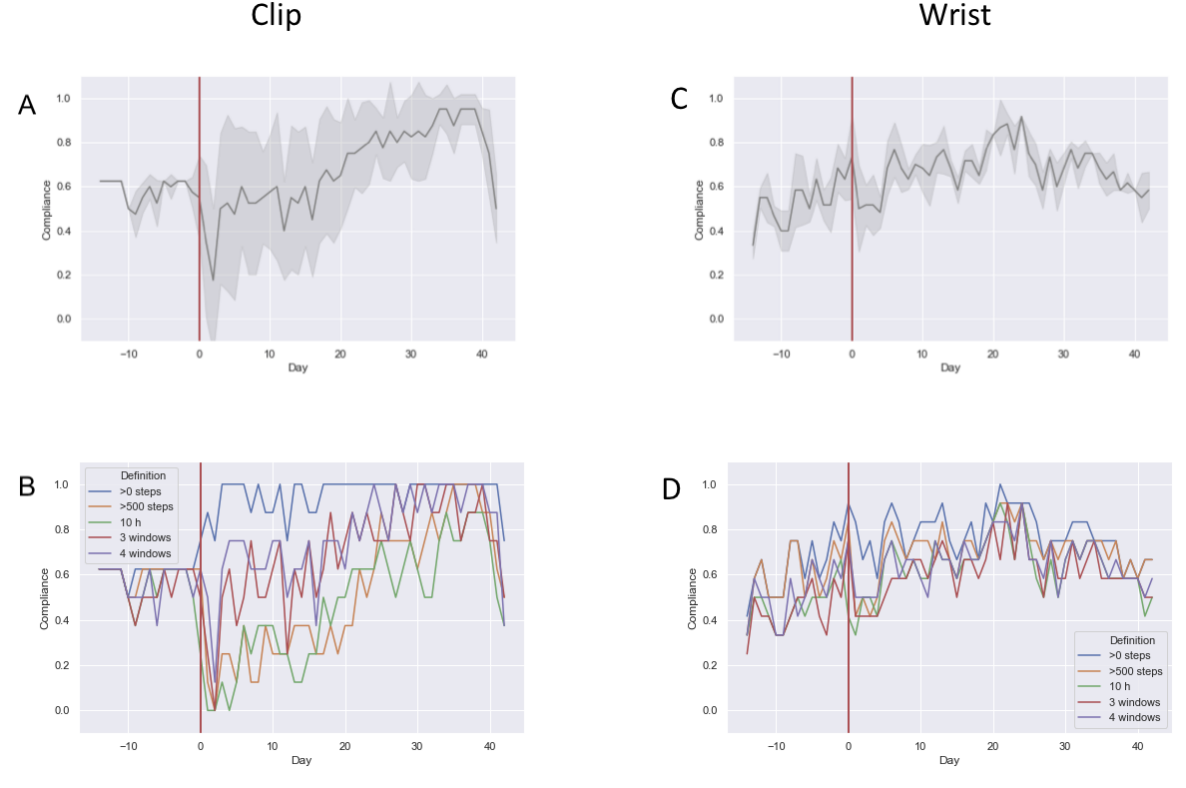
Temporal variations in compliance for the 5 criteria over the perioperative period. (A) The proportion of the clip-worn group that was compliant on each day. (B) The variation between criteria for the clip-worn group. (C) The proportion of the wrist-worn group that was compliant on each day. (D) The variation between criteria for the wrist-worn group.

Based on our earlier finding that deviation between measures corresponds to device type (wrist vs clip), we examined the temporal variations for each device independently. Figure 5A shows the proportion of the clip-worn group that was compliant on each day. It can be seen that there is close agreement between the criteria during the presurgery phase. At the point of surgery there is a noticeable increase in deviation between criteria, which gradually decreases over time.

Figure 5B again shows that compliance is particularly sensitive to criterion selection during the first 3-4 weeks of the postoperative period, with >0 steps close to 100% throughout. The >500 steps and 3-of-4 window outcomes are closely aligned during this phase, as are ≥10 hours and 3-a-day. However, the overall range is very large.

Figure 5C shows the proportion of the wrist-worn group that was compliant on each day. Contrasting Figure 5A (clip group) with Figure 5C (wrist group), we see that the wrist-worn group exhibit far lower deviation between measures throughout the entire period, and only a small increase in deviation immediately following surgery. For the wrist group, the deviation level remains consistently low from approximately 2 weeks postsurgery. Figure 5D also shows close agreement between criteria. This reveals that the deviation between measures is a more prominent issue for the clip device group, but that the issue only becomes apparent at the point of surgery.

To investigate these observations quantitatively, we compared the effects of *gender*, *age*, *BMI*, *stage*, and *device type* (*clip or wrist-worn tracker*) on the SD between compliance outcomes using linear mixed models. We again separated the perioperative period into 4 distinct 2-week stages. (The SDs for each patient in the 4 stages are shown in Multimedia Appendix 4.) ASA was again excluded. The independent variables were set up as fixed effects and ID was set as a random effect. The β estimates indicate the value by which SD between compliance outcomes changes.

Overall, we found a significant main effect of *stage* on SD between compliance outcomes, *F*_3,54_=11.421, *P*<.001. Deviation between outcomes was significantly higher (approximately 8%) in the first 2 weeks postsurgery (β=.082, SE=0.016, *P*<.001).

We also found a significant interaction effect between *device type* and *stage* (*F*_3,54_=9.976, *P*<.001). For wearers of the clip-on device, deviation between measures was significantly higher (approximately 6%) in the 2 weeks immediately following surgery (β=.064, SE=0.016, *P*<.001). These outcomes provide further evidence of how surgery appears to impact patients’ compliance, with reductions in activity in weeks 0-2 causing some patients to miss the requirements of stricter thresholds, resulting in a greater quantity of data being discarded. This effect is especially pronounced among those who wore the tracker as a clip-on device (Figure 5B).

## Discussion

### Principal Findings

The first finding of this study was that the 5 compliance criteria provide different compliance outcomes when applied to the patients’ data. We found a 24% difference in average rates of data retention between the most lenient criterion (>0 steps) and the most stringent (≥10 hours), with other measures falling between these 2 points. This finding illustrates that different compliance criteria cannot be used interchangeably to analyze data from TKA patients, and dovetails with the work of Tang et al [10], who observed similar effects after applying 4 criteria to 9 distinct activity tracking data sets. A unique feature of our work is the examination of TKA patients, who are experiencing temporary impairment to their mobility due to surgery. Our study reinforces the need to carefully consider an appropriate criterion for assessing compliance and shows that this is especially crucial when working with a surgery population, given impairments to their mobility.

Our second main finding was that differences in compliance outcomes were not uniform across the sample. The data of some patients were largely unaffected by the use of different criteria, whereas others varied considerably. The most extreme case resulted in 72% of a patient’s data being excluded when switching between 2 different criteria. Such a change would represent a clinically significant difference in the patient’s recorded activity level [17,39]. Our investigation of potential causes revealed that patients who wore the Fitbit as a clip-on had greater variation between compliance outcomes, compared with those who wore the device on their wrist. One explanation for this finding may be related to how the activity tracker is treated once it is in the home. Anecdotally, our patients mentioned attaching the clip-on Fitbit to their clothes and forgetting to remove it at the end of the day. Using wrist-worn trackers may therefore result in higher quality data because they are less likely to be forgotten by patients and because a wrist-worn device does not need to be removed as often (eg, while asleep).

The third main contribution from our study lies in demonstrating the impact of surgery on calculations of compliance. We found that patients exhibit changes in tracking behavior and fluctuations in physical activity after surgery. Statistical analyses also showed that the deviation between compliance outcomes was significantly higher (*P*<.001) in the 2 weeks following surgery. Specifically, compliance was calculated as significantly higher (*P*<.001) when using >0 steps, which is also the most lenient criterion. The implication of these findings is that analyses of compliance need to account for reductions in physical mobility following a surgical procedure. Stricter measures of compliance may exclude data on the basis of low activity, even though this activity may in fact be representative of the patient’s capabilities following surgery. Although the >0 steps criterion is vulnerable to recording incidental step data from arm movements [34], the presence of these “steps” may help to show the tracker was worn and may be preferable to discarding days entirely. This consideration is important for clinicians who plan to use physical activity data from TKA patients, and for systems designed to support decisions based on its use.

### Implications for Activity Tracking Studies

On the basis of our study, we suggest that compliance analysis among TKA patients should begin by using the >0 steps criterion as an initial data filter. This enables identification of days on which the tracker was worn, allowing noncompliant days to be removed from calculations of step count. Next, a windowed approach that has a minimal activity target, such as 3-of-4 windows [36], should be used to determine whether the patient recorded data across the day.

Regarding methodology, we echo Tang et al [10] and encourage future researchers to clearly describe the approach taken when calculating and reporting compliance. Researchers should also state the criterion used to determine a valid day and consider how other criteria may affect the data. Failure to describe the compliance measure may undermine the perceived validity of research using activity tracking data.

Finally, future studies should consider using secondary data sources to acquire ground truth about whether a patient was wearing an activity tracker. Many activity trackers now include heart rate monitors, and although these do not guarantee improvements in compliance [40], the additional data source could be triangulated with step counts to determine wear time. A limitation of these devices, however, is that they have a shorter battery life than trackers such as the Fitbit Zip, and hence may impact compliance because patients need to recharge the device [32]. Ecological momentary assessment [41] techniques, delivered via a participant’s smartphone, could also be used to collect self-reports about whether a tracker is being worn.

### Design Implications

The results of this study can inform presurgery and postsurgery monitoring systems (eg, [26]) that support clinical decision making on the basis of activity data. Our study showed that the underlying threshold used to calculate compliance can affect rates of data retention. Decisions made on the basis of these data may be flawed if the system excludes a large proportion of valid days due to notionally low activity. We suggest that systems should show the exact number of steps recorded and how these steps were distributed over the day, enabling clinicians to assess compliance at the absolute and temporal levels. Noncompliant days should be illustrated as gaps in the patient’s record, which may still be useful to support decision making [14] and encourage patients to increase their compliance with tracker use.

### Clinical Implications

Based on this study, clinical trials involving activity trackers must account for the fact that stringent compliance criteria may lead to the exclusion of potentially useful data from TKA patients. Selecting a lenient criterion can address this concern. Similarly, clinical monitoring systems (eg, [26]) will present unreliable data without an appropriate compliance measure.

Researchers should also be aware that patients undergoing TKA have a relatively high dropout rate in the context of use of wearables. Our study was conducted in the UK, where the waiting times for surgery mean there is a substantial time gap from being listed for surgery and the surgery itself. Trials should recognize the potential for patient forgetfulness if the activity trackers are handed out early in the patient journey. Likewise, trials should recognize that the postoperative period is often painful, with the consequent possibility for patients to be distracted from using the wearable as it may not be high on their list of priorities.

### Limitations

The number of analyzed participants is a limitation of this study. Our sample of 20 patients was sufficient to investigate differences and illustrate patterns but needs to be verified with larger sample sizes. A cohort of over 100 patients is likely to be needed to draw clinical conclusions about correlations between early activity, compliance, and other factors such as pain, analgesia use, and late outcomes. Knee replacement patients are known to have an approximate 20% dissatisfaction rate past 1 year [23]. Early activity may be a predictor in this likely multifactorial problem, and future work should seek higher statistical power to shine a light on this.

Another limitation is that, because of the open invitation for participation in the study, patients with greater concern about physical activity may have accepted the invitation. Rates of compliance with device use may be different among those who are less concerned about physical activity.

Lastly, our study was not designed to investigate differences between wearing the tracker on different parts of the body. This means that participants were not randomly assigned to wear the tracker in particular places, and thus we cannot completely rule out other latent factors as explanations for compliance differences between wrist-worn and clip-on trackers. Possible influences include gradual improvements in administering the study protocol, or changes in staff, at the collaborating hospital. Our analysis led us to explore demographic factors and the placement of the Fitbit as these were available to us based on the data we collected. Other factors that may impact patients’ behavior (eg, smoking status) should be included in future work.

### Conclusions

This study aimed to understand how different criteria affect calculated compliance with activity tracking in TKA patients. Our findings suggest that different compliance criteria cannot be used interchangeably to analyze activity data provided by patients following ambulatory surgery. Instead, reductions in postsurgery mobility necessitate the use of lenient compliance criteria, such as >0 steps, combined with a windowed approach. These criteria can account for temporary mobility impairments while also tracking wear over the course of a day. Encouraging patients to wear the device at their wrist, and using secondary sources of data as ground truth, can increase confidence in compliance outcomes by ensuring that activity is detected and by increasing patients’ actual wear time.

## Appendix

Multimedia Appendix 1 Participant Information Sheet.

Multimedia Appendix 2 Table 3. Comparison of five compliance criteria applied to the activity tracking data of 20 different patients. A person is considered to be compliant if they meet the criterion on a given day. The calculation is provided as a percentage of compliant days over the 8-week perioperative period.

Multimedia Appendix 3 Heatmaps for all 20 Participants.

Multimedia Appendix 4 Table 4: Mean and Std Dev comparison.

## Abbreviations

MKUH: Milton Keynes University Hospital
SE: standard error
TKA: total knee arthroplasty
