# Supplementary material for: Measuring Daily Compliance With Physical Activity Tracking in Ambulatory Surgery Patients: Comparative Analysis of Five Compliance Criteria
Source: JMIR Mhealth Uhealth. 2021 Jan 26;9(1):e22846. doi: 10.2196/22846 (PMC7872832; doi:10.2196/22846)
Supplement: Multimedia Appendix 1 [file mhealth_v9i1e22846_app1.pdf]

Milton Keynes University Hospital NHS
NHS Foundation Trust

The Open University

UNIVERSITY OF EXETER

A feasibility study to monitor post discharge activity of patient recovery after knee replacement surgery

Patient Information Sheet

We would like to invite you to take part in our research study. Before you decide we would like you to understand why the research is being done and what it would involve for you. We suggest that it may take about 10 minutes to read thoroughly. If, after reading this information sheet, you think you may be interested in taking part one of our team will go through the sheet with you via telephone and answer any questions you have.

You have been selected because you are a new patient who is scheduled to have knee replacement surgery, and at this stage there are approximately 50 patients expected to take part.

Summary

We know from previous research that it is important for people recovering from knee surgery to exercise after surgery, however not much is known about how much exercise is needed, and if there are critical times in the recovery process (e.g. soon after surgery) when people should exercise to get the most benefit.

The purpose of this study is to measure the number of steps taken daily by people recovering from knee surgery, and examine how this relates to the speed and extent of their recovery.

All we are requesting you do to is carry a pedometer with you at all times until your first consultation with your doctor post-surgery (approximately 6 weeks after surgery), and answer some questionnaires during your normal treatment sessions. The pedometer records the number of steps you take daily.

What's involved?

The study will be included in your normal treatment sessions at the outpatient orthopaedic clinic. At the first meeting before your surgery, if you provide your consent to participate in this study, you will be given instructions on the use of the pedometer, and we will ask you to complete a questionnaire.

Approximately 2 weeks before your surgery date we will send you a pedometer and a box to plug into your broadband router (this uses about 1p in electricity/month). You will be asked to carry the pedometer with you at all times you are active (except sleeping/bathing) over the study period. You will also be asked to complete a daily pain and medication diary or we may give you a small box to push a button on to indicate your pain level instead. There will be contact details included for you to phone someone if you need some help when setting this up or if you have any questions.

Your physiotherapist will meet with you in your standard treatment sessions, at which time you will be asked if you are still happy to participate in the study, and you may be asked to again complete some short questionnaires.

We will ask you for your email address so that we can send you an email reminder about the study once a week.

At the end of the study you will be emailed a summary of your activity (steps taken) over the 6 weeks. When you attend your consultant appointment approximately 6 weeks after surgery you will be asked to return the pedometer and USB device or synchronization box. If you forget we will send you a pre-paid envelope to return it.

Your treatment will not end when the study ends.

We would also like to ask you if you would give your consent to being contacted in the future where you may be asked to take part in another interview. The purpose of this interview(s) will be to ask you about your experience of using the pedometer during the study, and delve further based on your responses. We will also be asking you whether you would consider continuing using life logging technologies. This additional interview is not a condition of your participation of this study.

What are the potential benefits and risks?

There will be no remuneration from participating in this research.

You may find it useful and interesting to learn more about how much activity you are engaging in during your recovery process, but we cannot promise the study will immediately help you. The information we get from this study, however, will help improve the treatment of people recovering from knee surgery.

There are no foreseeable risks arising from participation in this research, although you may find it mildly inconvenient to remember to carry the pedometer with you.

What will happen if I don't want to carry on with the study?

Your participation in the study is entirely **voluntary**, and you may **discontinue at any time**, without prejudice. Please note however that we may not be able to withdraw your data once the research has been analysed and written up for publication. Withdrawing from the study will not affect your treatment. If you withdraw from the study we may wish to still use your data recorded up until that point, and in that case one of the members of the research team will contact you to ask for your consent to do so.

How will my information be kept confidential?

The data collected will only be accessible to the researchers, your doctor, to you, and to anyone you choose to share the data with. None of your data will be made publically available, and it will all be stored in electronic format that is secured with passwords.

It may not be possibly to keep your identity anonymous to the researchers, however personally identifying information will not be electronically stored with your data. Instead a unique ID will be generated to record your data and we will ensure that you can never be identified by any data we publish.

The data will be retained for up to 5 years after the study. The results of this study will be used mainly to develop a larger longer term study, but they may also be published in a scientific journal.

With your consent we will notify your GP that you are taking part in this study.

Who is organising and funding this study?

This research is in part being supported by a research grant from the Engineering and Physical Sciences Research Council, and researchers from the Open University and the University of Exeter. Your doctor will not be paid for including you in this study.

Who has reviewed this study?

All research in the NHS is looked at by independent group of people, called a Research Ethics Committee, to protect your interests. This study has been reviewed and given favourable opinion by the NRES Committee London - Surrey Borders (REC Reference: 15/LO/0649)

What if I experience problems?

If you have a concern about any aspect of this study, you can ask to speak to Mr Pearce, Consultant Orthopaedic Surgeon, Milton Keynes Hospital, in the first instance.

If you believe that you have been harmed in any way by taking part in this study, you have the right to pursue a complaint and seek any resulting compensation through Milton Keynes Hospital NHS Foundation Trust who are acting as the research sponsor. Details about this are available from the research team. Also, as a patient of the NHS, you have the right to pursue a complaint through the usual NHS process. To do so, you can submit a written complaint to the Patient Liaison Manager, Complaints Office

The complaints team
Milton Keynes Hospital NHS Foundation Trust
Standing Way
Milton Keynes
MK6 5LD
(Phone 01908 243633)
Email PALS@mkhospital.nhs.uk

Negligence

In the event that something does go wrong and you are harmed during the research and this is due to someone‘s negligence then you may have grounds for a legal action for compensation, either against The Open University if it is in connection with this particular study or against Milton Keynes Hospital if it is in connection with your treatment, but you may have to pay your legal costs. The normal National Health Service complaints mechanisms will still be available to you (if appropriate).

Invitation to ask further questions

Please feel free to speak to your doctor if you have any concerns or questions in relation to this study before signing this Consent Form. You may also ask any questions throughout and after completion of the study.

You can contact the researchers:

Avelie Stuart (University of Exeter) on 01392 746964 or a.stuart@exeter.ac.uk

Blaine Price (Open University) on 01908 653 701, or b.a.price@open.ac.uk

Or you can speak to your doctor or physiotherapist at the outpatient clinic.

Thank you for taking the time to read this information sheet and considering taking part in this study
