# Supplementary material for: Measuring Daily Compliance With Physical Activity Tracking in Ambulatory Surgery Patients: Comparative Analysis of Five Compliance Criteria
Source: JMIR Mhealth Uhealth. 2021 Jan 26;9(1):e22846. doi: 10.2196/22846 (PMC7872832; doi:10.2196/22846)
Supplement: Multimedia Appendix 2 [file mhealth_v9i1e22846_app2.docx]

Appendix 2 – Tables showing compliance calculations for the 20 patients in our sample.

Table 3. Comparison of five compliance criteria applied to the activity tracking data of 20 different patients. A person is considered to be compliant if they meet the criterion on a given day. The calculation is provided as a percentage of compliant days over the 8-week perioperative period.

|  |  |  |  |  |  | **Compliance Calculation** | | | | |  |
| --- | --- | --- | --- | --- | --- | --- | --- | --- | --- | --- | --- |
| **ID** | **Age** | **Gender** | **BMI** | **ASA** | **Device** | **> 0 Steps** | **> 500 Steps** | **>= 10 Hours** | **3-a-day** | **3-of-4** | **Std Dev** |
| 1 | 65 | F | 22.7 | 2 | Wrist | 1.00 | 0.98 | 0.96 | 0.98 | 0.96 | 0.01 |
| 2 | 60 | F | 33.5 | 1 | Wrist | 0.77 | 0.75 | 0.75 | 0.75 | 0.75 | 0.01 |
| 3 | 66 | F | 34.2 | 2 | Wrist | 0.61 | 0.61 | 0.58 | 0.60 | 0.60 | 0.02 |
| 4 | 68 | F | 45.1 | 2 | Wrist | 0.75 | 0.70 | 0.70 | 0.72 | 0.72 | 0.02 |
| 5 | 66 | F | 27 | 2 | Clip | 0.89 | 0.81 | 0.79 | 0.84 | 0.84 | 0.05 |
| 6 | 63 | F | 27 | 2 | Wrist | 1.00 | 1.00 | 0.98 | 0.89 | 0.96 | 0.05 |
| 7 | 63 | F | 46 | 2 | Wrist | 0.70 | 0.61 | 0.56 | 0.54 | 0.54 | 0.07 |
| 8 | 37 | F | 24.9 | 2 | Wrist | 0.49 | 0.42 | 0.33 | 0.33 | 0.33 | 0.08 |
| 9 | 72 | F | 29.5 | 2 | Wrist | 0.81 | 0.74 | 0.60 | 0.67 | 0.65 | 0.09 |
| 10 | 62 | M | 28 | 2 | Wrist | 1.00 | 0.98 | 0.82 | 0.84 | 0.88 | 0.09 |
| 11 | 73 | M | 25.3 | 2 | Clip | 0.72 | 0.61 | 0.49 | 0.70 | 0.68 | 0.10 |
| 12 | 66 | M | 33 | 2 | Clip | 0.89 | 0.72 | 0.63 | 0.70 | 0.72 | 0.11 |
| 13 | 67 | F | 39 | 2 | Wrist | 0.81 | 0.70 | 0.53 | 0.74 | 0.67 | 0.12 |
| 14 | 75 | F | 30 | 2 | Wrist | 0.74 | 0.53 | 0.40 | 0.46 | 0.47 | 0.15 |
| 15 | 50 | M | 32 | 2 | Clip | 0.70 | 0.35 | 0.37 | 0.61 | 0.61 | 0.18 |
| 16 | 67 | M | 27.7 | 2 | Wrist | 0.40 | 0.26 | 0.02 | 0.05 | 0.04 | 0.18 |
| 17 | 67 | M | 28.9 | 2 | Clip | 1.00 | 0.60 | 0.72 | 0.93 | 0.93 | 0.19 |
| 18 | 56 | F | 38 | 2 | Clip | 0.81 | 0.28 | 0.35 | 0.63 | 0.74 | 0.25 |
| 19 | 71 | F | 44.6 | 2 | Clip | 0.96 | 0.39 | 0.32 | 0.61 | 0.56 | 0.29 |
| 20 | 76 | M | 29 | 3 | Clip | 1.00 | 0.60 | 0.28 | 0.82 | 0.67 | 0.31 |
