# Supplementary figures and images for: Measuring Daily Compliance With Physical Activity Tracking in Ambulatory Surgery Patients: Comparative Analysis of Five Compliance Criteria
Source: JMIR Mhealth Uhealth. 2021 Jan 26;9(1):e22846. doi: 10.2196/22846 (PMC7872832; doi:10.2196/22846)

# Appendix 3: Heatmaps for all 20 Participants

| P01  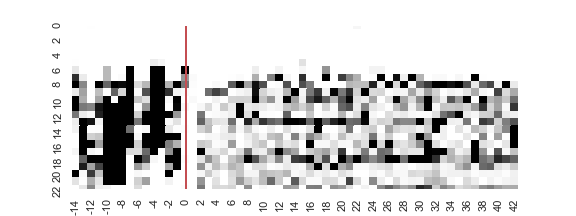 | P11  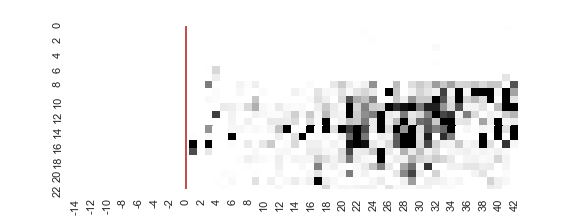 |
| --- | --- |
| P02  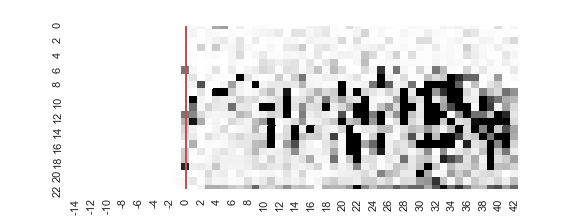 | P12  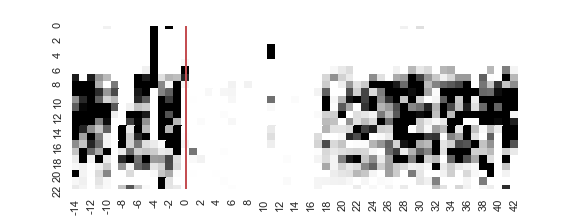 |
| P03  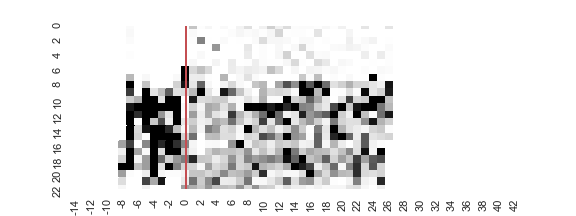 | P13  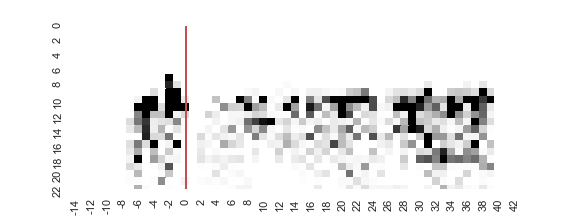 |
| P04  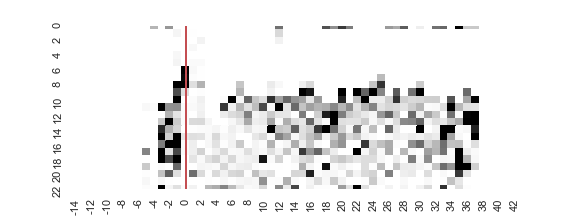 | P14  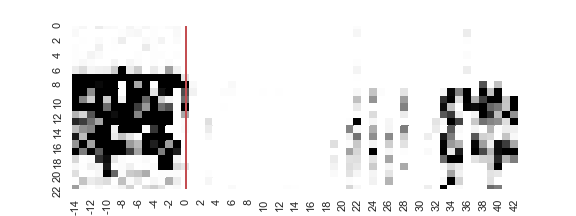 |
| P05  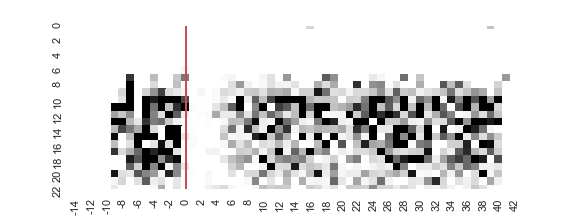 | P15  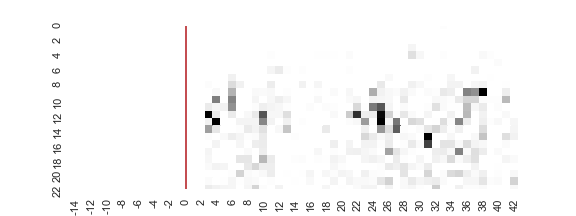 |
| P06  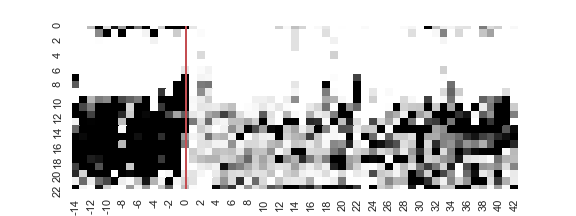 | P16  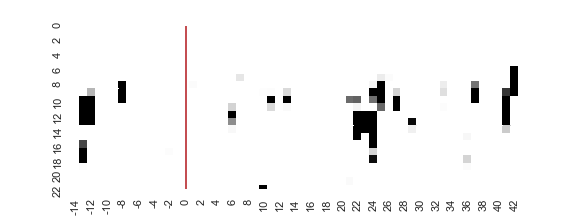 |
| P07  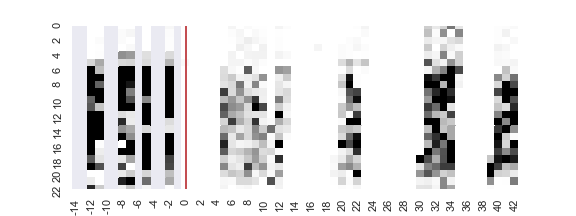 | P17  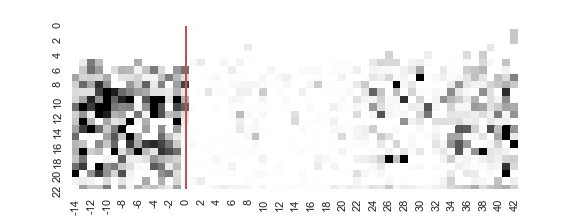 |
| P08  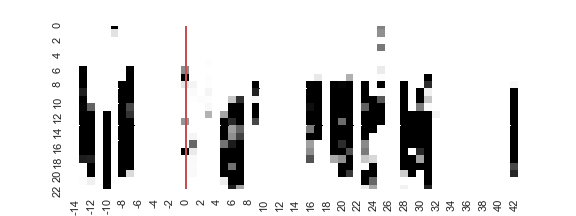 | P18  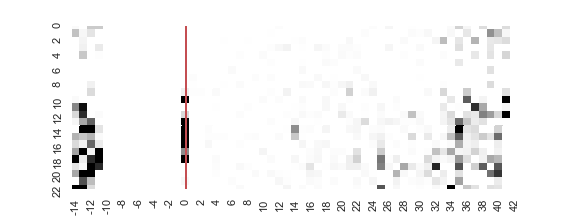 |
| P09  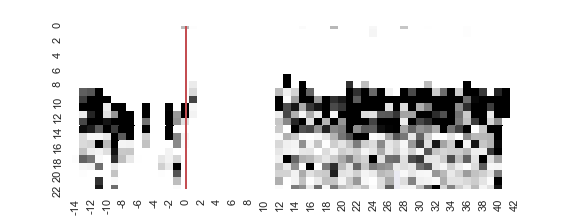 | P19  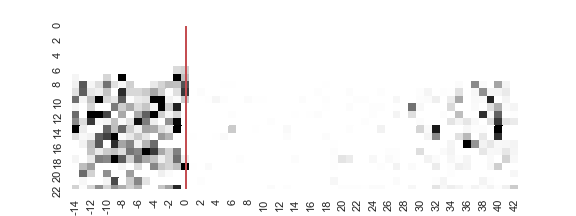 |
| P10  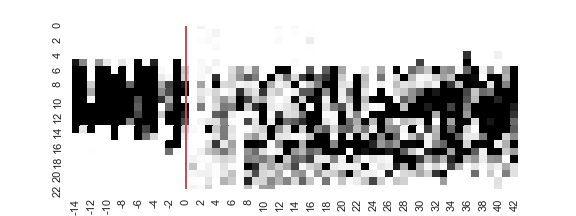 | P20  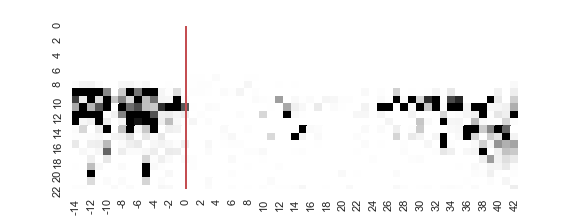 |

Supplement: Multimedia Appendix 3 [file mhealth_v9i1e22846_app3.docx]
