# Supplementary material for: Measuring Daily Compliance With Physical Activity Tracking in Ambulatory Surgery Patients: Comparative Analysis of Five Compliance Criteria
Source: JMIR Mhealth Uhealth. 2021 Jan 26;9(1):e22846. doi: 10.2196/22846 (PMC7872832; doi:10.2196/22846)
Supplement: Multimedia Appendix 4 [file mhealth_v9i1e22846_app4.docx]

Appendix 4 – Comparison of mean and standard deviation of compliance criteria.

Table 4. Comparison of mean and standard deviation of compliance criteria, separated into two-week intervals.

|  | **Pre-Surgery** | **Surgery Day** | **Week 0-2** | **Week 2-4** | **Week 4-6** |
| --- | --- | --- | --- | --- | --- |
| >0 Steps | 0.625 | 0.85 | 0.84285714 | 0.88571429 | 0.82857143 |
| >500Steps | 0.59642857 | 0.75 | 0.48928571 | 0.66785714 | 0.74285714 |
| >= 10hours | 0.50714286 | 0.35 | 0.43571429 | 0.63928571 | 0.64642857 |
| 3-a-day | 0.48571429 | 0.65 | 0.53214286 | 0.72142857 | 0.70357143 |
| 3-of-4 windows | 0.53214286 | 0.7 | 0.61071429 | 0.75357143 | 0.74285714 |
| MEAN | 0.54928571 | 0.66 | 0.58214286 | 0.73357143 | 0.73285714 |
| STDEV | 0.05304022 | 0.18841444 | 0.15917918 | 0.09605085 | 0.06649958 |
